# Supplementary material for: Perinatal Acetaminophen Exposure and Childhood Attention-Deficit/Hyperactivity Disorder (ADHD): Exploring the Role of Umbilical Cord Plasma Metabolites in Oxidative Stress Pathways
Source: Brain Sci. 2021 Sep 30;11(10):1302. doi: 10.3390/brainsci11101302 (PMC8533963; doi:10.3390/brainsci11101302)
Supplement: Supplementary file 1 [file brainsci-11-01302-s001.zip › brainsci-1375082-supplementary.pdf]

Supplementary materials for “Perinatal acetaminophen exposure and childhood attention-deficit/hyperactivity disorder (ADHD): exploring the role of umbilical cord plasma metabolites in oxidative stress pathways”

Figure S1. Association of cord plasma unmetabolized acetaminophen[a] with childhood ADHD[b,c]

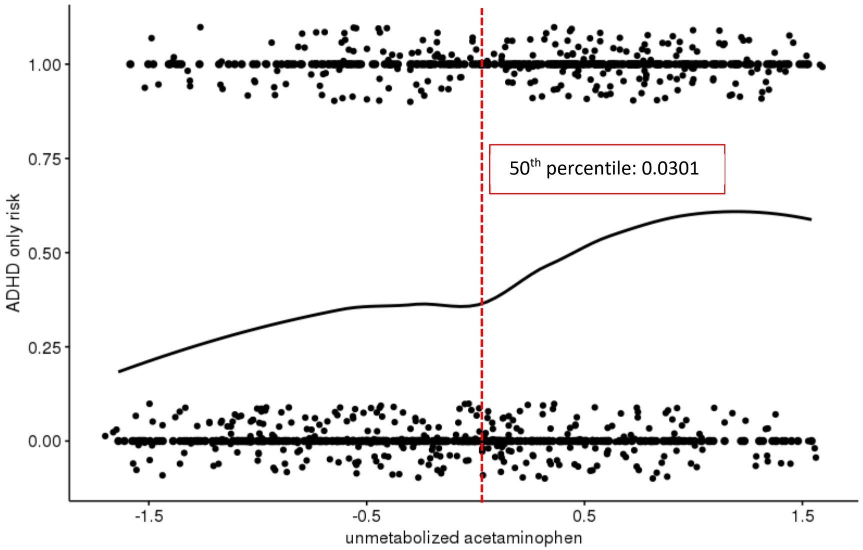

[a] Inverse normal transformed intensities: 5th to 95th percentile
[b] Reference group = neurotypical development

Table S1. Odds of childhood ADHD[a] stratified by cord plasma unmetabolized acetaminophen and 8-hydroxy-deoxyguanosine[b] ≤50th and >50th percentile

	Total				Males				Females			
	N	Odds Ratio	95% CI	P-value	*N*	Odds Ratio	95% CI	P-value	*N*	Odds Ratio	95% CI	P-value
cord acetaminophen ≤50th percentile												
cord 8-hydroxydeoxyguanosine ≤50th percentile	148	1.00 (ref)	–	–	81	1.00 (ref)	–	–	67	1.00 (ref)	–	–
cord 8-hydroxydeoxyguanosine >50th percentile	136	1.10	(0.67, 1.80)	0.698	69	0.94	(0.49, 1.79)	0.849	67	1.86	(0.76, 4.76)	0.180
cord acetaminophen >50th percentile												
cord 8-hydroxydeoxyguanosine ≤50th percentile	136	2.21	(1.37, 3.58)	0.001*	69	2.21	(1.17, 4.25)	0.016*	57	2.74	(1.13, 7.01)	0.029*
cord 8-hydroxydeoxyguanosine >50th percentile	148	2.38	(1.49, 3.82)	<0.001*	86	2.98	(1.57, 5.79)	0.001*	62	2.24	(0.92, 5.73)	0.080

[a] Reference group = neurotypical development; ADHD only: children with only a diagnosis of attention-deficit hyperactivity disorder

[b] Inverse normal transformed intensities

*p <0.05

Table S2. Adjusted logistic regressions examining risk of childhood ADHD for cord plasma metabolites stratified by sex[a,b]

	Males N=315 (124 neurotypical, 191 ADHD only)			Females N=253 (196 neurotypical, 57 ADHD only)		
	Odds Ratio	95% CI	P-value	Odds Ratio	95% CI	P-value
**ADHD only**[a]						
regressions for individual metabolites[c]						
acetaminophen >50th percentile[d]	2.60	(1.57, 4.36)	<0.001*	1.41	(0.74, 2.68)	0.295
methionine	1.33	(1.03, 1.72)	0.031*	1.83	(1.26, 2.74)	0.002*
glycine	1.29	(1.01, 1.65)	0.040*	1.67	(1.20, 2.38)	0.003*
serine	1.26	(0.98, 1.64)	0.079*	1.38	(0.99, 1.95)	0.062
glutamate	1.04	(0.81, 1.34)	0.740	1.55	(1.10, 2.22)	0.013*
8-hydroxy-deoxyguanosine	1.29	(1.00, 1.70)	0.057	1.21	(0.86, 1.68)	0.265
regression with both acetaminophen and methionine[c]						
acetaminophen >50th percentile[d]	2.41	(1.41, 4.19)	0.001*	1.11	(0.55, 2.23)	0.759
methionine	1.33	(0.90, 1.98)	0.150	2.23	(1.26, 4.20)	0.009*
regression with both acetaminophen and serine[c]						
acetaminophen >50th percentile[d]	2.48	(1.45, 4.29)	0.001*	1.21	(0.62, 2.36)	0.581
serine	1.22	(0.82, 1.82)	0.334	1.81	(1.04, 3.26)	0.041*
regression with both acetaminophen and glycine[c]						
acetaminophen >50th percentile[d]	2.40	(1.40, 4.16)	0.002*	1.15	(0.57, 2.31)	0.701
glycine	1.22	(0.84, 1.78)	0.298	2.85	(1.55, 5.76)	0.002*
regression with both acetaminophen and glutamate[c]						
acetaminophen >50th percentile[d]	2.65	(1.58, 4.49)	<0.001*	1.18	(0.59, 2.32)	0.632
serine	0.99	(0.69, 1.42)	0.955	1.49	(0.91, 2.51)	0.118
regression with both acetaminophen and 8-hydroxy-deoxyguanosine [c]						
acetaminophen >50th percentile[d]	2.57	(1.54, 4.32)	<0.001*	1.36	(0.70, 2.63)	0.364
8-hydroxy-deoxyguanosine	1.26	(0.86, 1.85)	0.244	1.11	(0.67, 1.81)	0.684

[a] Reference group = neurotypical development; ADHD only: children with only a neurodevelopmental diagnosis of attention-deficit hyperactivity disorder

[b] Adjusted model covariates: maternal age at delivery in years, parity (nulliparous vs. multiparous), maternal race/ethnicity (Black, white, Hispanic, or other), maternal education level (below college degree vs. above college degree), maternal body mass index (BMI), stress during pregnancy (mild, average, severe), maternal fever during pregnancy (yes, no), smoking during pregnancy (never, quit,

or continuous), alcohol use before or during pregnancy, marital status (not married vs. married), child sex, delivery type (cesarean vs. vaginal), preterm birth (<37 weeks), and low birthweight (<2500g)
[c] Inverse normal transformed intensities
[d] Reference group = cord acetaminophen level ≤ 50th percentile
*p <0.05

Table S3. Analysis of cord plasma methionine and glycine as mediators for the association between cord plasma unmetabolized acetaminophen and childhood ADHD diagnosis[a,b]

	Natural indirect effect [d] (log odds ratio)	SE	P-value	Total effect of cord acetaminophen[d] (log odds ratio)	% Mediated
**methionine[d]**					
Cord acetaminophen >50th percentile[c]	0.167	0.071	0.019*	0.758	22.1%
Cord acetaminophen as continuous	0.081	0.038	0.030*	0.403	18.9%
**glycine[d]**					
Cord acetaminophen >50th percentile[c]	0.166	0.078	0.032*	0.757	22.0%
Cord acetaminophen as continuous	0.079	0.038	0.040*	0.429	18.4%

[a] Mediation analysis was performed with the VanderWeele-Vansteelandt approach utilizing the R package'medflex.

[b] Adjusted model covariates: maternal age at delivery in years, parity (nulliparous vs. multiparous), maternal race/ethnicity (Black, white, Hispanic, or other), maternal education level (below college degree vs. above college degree), maternal body mass index (BMI), stress during pregnancy (mild, average, severe), maternal fever during pregnancy (yes, no), smoking during pregnancy (never, quit, or continuous), alcohol use before or during pregnancy, marital status (not married vs. married), child sex, delivery type (cesarean vs. vaginal), preterm birth (<37 weeks), and low birthweight (<2500g)

[c] Reference group = cord acetaminophen level ≤ 50th percentile

[d] Inverse normal transformed intensities

*p <0.05

Table S4. Sensitivity analyses for cord plasma methionine and glycine as a partial mediators of the association between cord plasma unmetabolized acetaminophen and childhood ADHD diagnosis[a,b]

		methionine[c]					glycine[c]				
**Subgroup**	**N**	**Natural indirect effect (log odds ratio)**	**SE**	**P-value**	**Total effect of cord acetaminophen[c] >50th percentile (log odds ratio)**	**% Mediated**	**Natural indirect effect (log odds ratio)**	**SE**	**P-value**	**Total effect of cord acetaminophen[c] >50th percentile (log odds ratio)**	**% Mediated**
Excluding preterm births	490	0.161	0.082	0.049*	0.671	23.9%	0.132	0.091	0.149	0.671	19.6%
Excluding maternal alcohol use before or during pregnancy	535	0.150	0.079	0.056	0.799	18.8%	0.150	0.083	0.071	0.799	18.8%
Excluding maternal smoking (quit or continuous)	484	0.203	0.082	0.013*	0.701	29.0%	0.211	0.094	0.025*	0.701	30.1%
Excluding maternal fever during pregnancy	541	0.238	0.088	0.007*	0.784	30.3%	0.234	0.091	0.010*	0.785	29.8%

[a] Mediation analysis was performed with the VanderWeele-Vansteelandt approach utilizing the R package'medflex.

[b] Adjusted model covariates: maternal age at delivery in years, parity (nulliparous vs. multiparous), maternal race/ethnicity (Black, white, Hispanic, or other), maternal education level (below college degree vs. above college degree), maternal body mass index (BMI), stress during pregnancy (mild, average, severe), maternal fever during pregnancy (yes, no), smoking during pregnancy (never, quit, or continuous), alcohol use before or during pregnancy, marital status (not married vs. married), child sex, delivery type (cesarean vs. vaginal), preterm birth (<37 weeks), and low birthweight (<2500g).

[c] Inverse normal transformed intensities

*p ≤ 0.05

Table S5. Logistic regressions examining risk of childhood ADHD for cord plasma unmetabolized acetaminophen >50th percentile and maternal plasma methionine

		**Unadjusted Logistic**			**Adjusted Logistic**[e]		
	N[d]	Odds Ratio	95% CI	P-value	Odds Ratio	95% CI	P-value
**ADHD only**[a]	449						
regressions for individual metabolites[b]							
cord acetaminophen >50th percentile[c]		2.18	(1.48, 3.23)	<0.001*	1.94	(1.23, 3.09)	0.005
maternal methionine		0.97	(0.79, 1.17)	0.725	1.02	(0.80, 1.29)	0.894
maternal glycine		0.95	(0.78, 1.16)	0.628	0.92	(0.72, 1.17)	0.490
regression with both acetaminophen and methionine[b]							
cord acetaminophen >50th percentile[c]		2.18	(1.48, 3.24)	<0.001*	1.96	(1.23, 3.12)	0.004*
maternal methionine		1.00	(0.82, 1.22)	0.970	1.05	(0.82, 1.33)	0.715
regression with both acetaminophen and glycine[b]							
cord acetaminophen >50th percentile[c]		2.14	(1.45, 3.17)	0.001*	1.93	(1.22, 3.07)	0.005*
maternal glycine		0.97	(0.79, 1.19)	0.753	0.93	(0.73, 1.19)	0.581

[a] Reference group = neurotypical development; ADHD only: children with only a diagnosis of attention-deficit hyperactivity disorder;
[b] Inverse normal transformed intensities
[c] Reference group = cord acetaminophen level ≤ 50th percentile
[d] Sample size by neurodevelopmental diagnosis category: ADHD only – 164; neurotypical development – 285
[e] Adjusted model covariates: maternal age at delivery in years, parity (nulliparous vs. multiparous), maternal race/ethnicity (Black, white, Hispanic, or other), maternal education level (below college degree vs. above college degree), maternal body mass index (BMI), stress during pregnancy (mild, average, severe), maternal fever during pregnancy (yes, no), smoking during pregnancy (never, quit, or continuous), alcohol use before or during pregnancy, marital status (not married vs. married), child sex, delivery type (cesarean vs. vaginal), preterm birth (<37 weeks), and low birthweight (<2500g)
*p <0.05
